# Two Decades of BSAC Resistance Surveillance

**DOI:** 10.1093/jac/dkaf303

**Published:** 2025-10-27

**Authors:** James C Hurley, Alan P Johnson

**Affiliations:** Melbourne Medical School, University of Melbourne, Parkville, Victoria, Australia; Ballarat Clinical School, Deakin University, Ballarat, Victoria, Australia; Internal Medicine Service, Ballarat Health, Grampians Health Services, Ballarat, Victoria, Australia; Journal of Antimicrobial Chemotherapy, Birmingham, UK

This Supplement comprises a series of papers that analyse and summarize the outputs of the British Society for Antimicrobial Chemotherapy Antimicrobial Resistance Surveillance Project over its 20-year lifespan from 1999 to 2019. Its publication coincides with the 50th anniversary of the *Journal of Antimicrobial Chemotherapy*, which first appeared in 1975.

The introductory article by MacGowan *et al*. provides an overview of the establishment and achievements of the Project, with reference to other surveillance activities, such as the PHLS (now the UKHSA) national surveillance and the Alexander Project. Separate Programmes within the overall Project collected and assessed the antibiotic susceptibility of community- and hospital-acquired respiratory and bloostream infection isolates. As outlined in other papers in the series, the Project encompassed aspects beyond antimicrobial resistance, including the serotype distributions of *Streptococcus pneumoniae* isolates from bacteraemia or community associated lower respiratory tract infections. The Project's results, including the characterization of critical resistance mechanisms, provide robust and reliable data on the evolution of antimicrobial resistance in the UK and Ireland, based on targeted surveillance information from almost 100,000 isolates. The papers appearing here also highlight many previous publications arising from the Project that have appeared in this Journal and elsewhere since 2001.

Although the BSAC Surveillance Project ended in 2019, the data and the large collection of highly-characterized isolates, many of which have been sequenced, are archived at the University of Dundee, in collaboration with the University of St Andrews, and will remain available to researchers. Details of how to access the collection are included in the Supplement's final paper by Parcell *et al.*

The Editors hope that the results of this Project will serve as a bench-mark against which to assess the future evolution of antimicrobial resistance in the UK and Ireland, and that the Project's design will serve as a model for similar surveillance studies elsewhere in the world.

## Funding

This paper was published as part of a supplement supported by the British Society for Antimicrobial Chemotherapy.

## Transparency declarations

The authors are both members of BSAC but have no other conflicts to declare.

